# Validation of ICD‐11 PTSD and complex PTSD in foster children using the International Trauma Questionnaire

**DOI:** 10.1111/acps.13100

**Published:** 2019-10-16

**Authors:** A. Haselgruber, K. Sölva, B. Lueger‐Schuster

**Affiliations:** ^1^ Unit of Psychotraumatology Faculty of Psychology University of Vienna Vienna Austria

**Keywords:** Post‐traumatic stress disorder, complex PTSD, ICD‐11, foster children, International Trauma Questionnaire

## Abstract

**Objective:**

ICD‐11 introduces post‐traumatic stress disorder (PTSD) and complex PTSD (CPTSD) as two distinct trauma‐related disorders. Using the International Trauma Questionnaire (ITQ) as disorder‐specific measure, this study is the first to examine the factorial and construct validity of ICD‐11 PTSD, CPTSD and the ITQs’ applicability in children.

**Methods:**

Two hundred and eight Austrian foster children completed a set of standardized measures. Excluding participants who reported not having experienced any kind of trauma, a final sample of 136 children completed the ITQ. Factorial and construct validity of ICD‐11 CPTSD and psychometric properties of ITQ scales were assessed by factor analysis and latent class analysis.

**Results:**

Confirmatory factor analysis supported the two‐factor higher‐order model of ICD‐11 CPTSD in children by high factor loadings and excellent model fit. Reliability and regression analysis evidenced psychometric adequacy and discriminant validity of ITQ scales. Latent class analysis substantiated construct validity of ICD‐11 CPTSD, identifying a CPTSD (22.8%), PTSD (31.6%) and low symptoms class (45.6%). The CPTSD class showed highest rates of childhood trauma, comorbid psychopathology and functional impairment.

**Conclusion:**

Factorial and construct validity of ICD‐11 CPTSD was evidenced in children for the first time using precise descriptions of ICD‐11 symptom content, supporting the reliability and validity of the ITQ in children.

**Significant outcomes**

- Factorial validity of ICD‐11 CPTSD evidenced in children for the first time, supporting the distinction of PTSD and DSO as related but separate constructs.
- The International Trauma Questionnaires’ applicability in children was supported by the good psychometric properties and discriminant validity of its scales.
- Construct validity of ICD‐11 CPTSD was confirmed, associating CPTSD (22.8%) with higher rates of childhood trauma, psychopathology and functional impairment than PTSD (31.6%) and low symptoms (45.6%).

**Limitations**

- Results are based on a small sample of foster children, limiting the findings’ generalizability.
- Findings may deviate from true population effects due to possible underreporting of childhood trauma.
- No additional PTSD measure was used, not allowing to examine the findings’ concurrent validity.

## Introduction

With the recent publication of the 11th version of the International Classification of Diseases (ICD‐11), the World Health Organisation 1 introduced two distinct trauma‐related disorders under the general parent category ‘Disorders specifically associated with stress’: post‐traumatic stress disorder (PTSD) and complex PTSD (CPTSD). PTSD consists of three symptom clusters, including re‐experiencing the trauma here and now (Re), avoidance of traumatic reminders (Av) and persistent sense of current threat, manifesting in startle and hypervigilance (Th). CPTSD consists of the PTSD symptom clusters and additionally disturbances in self‐organization (DSO). DSO consists of three symptom clusters, including affective dysregulation (AD), negative self‐concept (NSC) and disturbances in relationships (DR). The symptom structure of CPTSD according to ICD‐11 is reflected in a multidimensional and hierarchical model, comprising PTSD and DSO as two distinct but related higher‐order factors.

In a number of factor‐analytic studies, this two‐factor higher‐order model was examined along alternative models, testing the symptom structure and factorial validity of ICD‐11 CPTSD. In the majority of studies, the two‐factor higher‐order model yielded the best fit across different samples 2, 3, 4, 5, 6, 7, 8. However, not all studies replicated these findings 9, 10. Despite extensive research in adult populations, to date no studies investigated the symptom structure of ICD‐11 CPTSD in children and adolescents (hereafter referred to as ‘children’ unless otherwise specified). Research on DSM‐5 PTSD in different age groups yielded a similar symptom structure in children and adults 11, 12, 13, providing evidence that this may also be the case for ICD‐11 PTSD and CPTSD. Examining the factorial validity of ICD‐11 PTSD and CPTSD in children is highly important, as it comprises a number of practical implications for assessment and treatment 14, 15.

Following the recent publication of the International Trauma Questionnaire (ITQ) 8, a validated instrument to assess ICD‐11 CPTSD in adults has become available. Despite this development, there are currently no measures to assess ICD‐11 CPTSD in children. Former studies in children used archival data 16 with inherent limitations of less precise formulations of ICD‐11 content, evidencing the need for an instrument to assess CPTSD in children.

Regarding construct validity, researchers have investigated whether the ICD‐11 conceptualization of CPTSD in fact describes a class of individuals that is distinctly different from individuals with PTSD by having a more ‘complex’ symptom profile with a higher number of clinically elevated symptoms 17. A number of latent class and latent profile analyses have supported this distinction. Studies in adults reported three‐ to four‐class solutions with a CPTSD class (high in PTSD and DSO symptoms), a PTSD class (high in PTSD and low in DSO symptoms), a low symptoms class (low in PTSD and DSO symptoms) and occasionally a DSO class (low in PTSD and high in DSO symptoms) 18, 19, 20, 21, 22, 23, 24. Similar to research on factorial validity, studies on construct validity of ICD‐11 CPTSD in children are scarce. The only study conducted in children to date reported a two‐class solution with a CPTSD and a PTSD class 16.

In line with the theoretical assumption that CPTSD is associated with higher rates of traumatization and greater number of clinically elevated symptoms 17, 25, symptom profiles of CPTSD in adult populations were repeatedly associated with significantly higher rates of traumatization 18, 19, 20, comorbidity 20, 22, 23 and functional impairment 19 than profiles of PTSD or low symptoms. In the only study to date conducted in a clinical sample of children, Sachser et al. 16 found that CPTSD was associated with higher rates of interpersonal trauma than PTSD, with no further differences regarding trauma history or psychopathology emerging. Despite these valuable first insights, the study was limited as it used archival data of measures that were designed to capture PTSD according to DSM‐based models of PTSD and assessed DSO using selected items from different trauma measures. Since ICD‐11 CPTSD contains not a mere subset of DSM‐5 PTSD symptoms and no measure to assess DSO was available at that point in time, these results should be replicated capturing the content aspects of ICD‐11 CPTSD precisely 3.

As a population of children that exhibit high rates of trauma exposure and a scale of mental health problems that is exceptional for a non‐clinical population 26, foster children are remarkably under‐investigated 27. A history of maltreatment by parental caregiver is the most common background for foster care placement, often involving substantiated experiences of abuse or neglect 28, 29. The majority of children in foster care experience sustained, repeated or multiple forms of childhood trauma (cumulative childhood trauma) 27, associated with increased risk to develop CPTSD 19. Accordingly, foster children exhibit significantly higher rates of PTSD and comorbid disorders than the general population 30, 31, 32 and it has been argued that these children exhibit a form of complex psychopathology that cannot be captured accurately using DSM‐5 or ICD‐10 classifications 26. Despite these issues and a cumulation of risk factors for the development of complex trauma‐related disorders, children in foster care are rarely investigated, and to our knowledge, no study examined the validity of complex trauma‐related disorders in this vulnerable population of children.

### Aims of the study

Deriving from the current state of knowledge, the aims of the present study are to (i) test the factorial validity of ICD‐11 CPTSD in children using the ITQ, (ii) assess the psychometric properties and discriminant validity of ITQ scales and (iii) test the construct validity of ICD‐11 CPTSD in children. Addressing aim (i), we hypothesized that the two‐factor higher‐order model would show the best model fit in our sample. Addressing aim (ii), that ITQ scales would show satisfactory internal reliability and exhibit discriminant validity. Addressing aim (iii), that distinct classes of individuals with symptom profiles reflecting CPTSD, PTSD and low symptoms would emerge and that these classes would differ regarding rates of childhood trauma, comorbid disorders and symptoms, and impairment in different domains.

## Methods

### Participants and procedures

Data used in this study were assessed in the course of a research project commissioned and financed by the government of Lower Austria. Assessments were conducted in six foster care facilities in Lower Austria, centrally managed by the government. All children currently living in foster care were invited to participate in the study voluntarily. Inclusion criteria for participation were as follows: age between 10 and 18 years, sufficient German language skills, stable mental health status (i.e. no psychotic states or heavy intoxication) and anticipated residence in long‐term care (i.e. longer than 12 months).

Between May and December 2018, 208 children participated in the study and completed a set of standardized measures. Assessments were conducted in the respective foster care facility by a team of trained clinical psychologists and trained master students in clinical psychology. Since maltreatment in childhood has been associated with impaired cognitive functioning and developmental delays 33, the administration of questionnaires was monitored closely to ensure their comprehension. Generally, group sessions were held with two children and one clinical psychologist, assisting children in filling out the questionnaires and answering any questions arising. If indicated due to cognitive, emotional or other reasons, interviews were conducted in private face‐to‐face sessions instead. Participation was voluntarily and written consent was obtained by each participant. The study was approved by the ethical board of the University of Vienna (#00328).

From the 208 children participating in the study, 20 had to be excluded because of large amounts of missing data (> 50% missings on the ITQ), and 52 reported not having experienced any kind of trauma and therefore did not fill out the ITQ, resulting in a final sample of 136 children with sufficient data on the ITQ. Excluded participants due to not having experienced trauma according to the self‐report did differ from included participants regarding gender (χ^2^ (1) = 8.934, *P *<* *0.05), age (*t* (185) = −2.106, *P *<* *0.05) and the tendency to minimize childhood trauma (χ^2^ (1)  = 9.579, *P *<* *0.05). In comparison, excluded participants were predominantly male (80.0% vs. 57.4%), marginally younger than included participants (*M* = 13.45, SD = 2.52 vs. *M* = 14.28, SD = 2.25), and a greater proportion showed the tendency to minimize childhood trauma (73.5% vs. 47.5%).

The mean age of the final sample was 14.28 years (SD = 2.25) with less females (42.6%) than males. The majority was born in Austria (87.5%) and currently went to special needs school (38.7%), secondary school (32.4%) or work‐related school (14.7%). The majority of children had contact with their parents (94.1%) and saw them on a weekly basis (74.3%). The mean time of foster care placement was 2.87 years (SD = 2.39).

### Measures

The International Trauma Questionnaire (ITQ) 8 is a 18‐item self‐report measure to assess ICD‐11 PTSD and CPTSD in adults. In the present study, the adult version of the ITQ was used and examined. Six items represent the three clusters of PTSD: Re (Re1, Re2), Av (Av1, Av2) and Th (Th1, Th2), and six items represent the three clusters of DSO: AD (AD1, AD2), NSC (NSC1, NSC2) and DR (DR1, DR2). Additionally, there are three items measuring functional impairment (social, occupational and other important areas of life) for the PTSD and the DSO clusters. Respondents indicate how much they were bothered by each symptom over the past month on a 5‐point Likert scale ranging from 0 (‘not at all’) to 4 (‘extremely’). Scores ≥ 2 (‘moderately’) indicate the presence of a symptom. PTSD diagnosis requires endorsement of one symptom in each PTSD cluster and associated functional impairment. CPTSD diagnosis requires a PTSD diagnosis, one symptom in each DSO cluster and associated functional impairment.

The Childhood Trauma Questionnaire (CTQ) 34 is a 28‐item measure to assess interpersonal childhood trauma and minimization of childhood trauma in children and adults. Each item is scored on a 5‐point Likert scale ranging from 1 (‘never true’) to 5 (‘very true’). Using provided cut‐off scores 35, the experience of different trauma types and cumulative childhood trauma (experience of more than one type) was assessed. The total CTQ score was used as indicator for overall childhood trauma. Higher scores reflect higher rates of traumatization. Reliability was good to excellent in the current study for emotional abuse (α = 0.89), physical abuse (α = 0.87), sexual abuse (α = 0.92) and emotional neglect (α = 0.83), only physical neglect (α = 0.46) was weak, as reported previously for the German version 36.

The Patient Health Questionnaire‐9 (PHQ‐9) 37 and the Generalized Anxiety Disorder Scale‐7 (GAD‐7) 38 were used to assess DSM‐IV major depressive disorder (MDD) (PHQ‐9) and generalized anxiety disorder (GAD) (GAD‐7). Respondents indicate how much they were bothered by each symptom over the past two weeks. Each item is scored on a 4‐point Likert scale ranging from 0 (‘not at all’) to 3 (‘nearly every day’). Scores ≥ 10 are used as cut‐off to identify diagnosis of MDD and GAD. The PHQ‐9 and the GAD‐7 have been frequently used in children with strong psychometric properties 39, 40, 41. Reliability of the PHQ‐9 (α = 0.85) and GAD‐7 (α = 0.89) was good in the current study.

The Adolescent Dissociative Experience Scale‐8 (ADES‐8) 42 is a 8‐item measure to assess dissociative symptoms in children. Respondents indicate how frequently they experience dissociative symptoms described on a numerical 11‐point scale ranging from 0 to 10. Higher scores reflect higher rates of dissociation, and scores ≥ 3 are used as cut‐off to identify clinically relevant dissociative symptoms. Reliability of the ADES‐8 was good in the current study (α = 0.84).

The Child Behaviour Checklist Youth Self‐Report Form (YSR 11‐18R) 43 is a 118‐item measure to assess children's social competence and behavioural problems. Respondents indicate how strongly they agree with each item on a 3‐point Likert scale ranging from 1 (‘not true’) to 3 (‘very true or often true’). The CBCL comprises eight syndrome scales and two second‐order scales. The second‐order scales for internalizing behaviour problems (α = 0.93) with 31 items and externalizing behaviour problems (α = 0.88) with 32 items were used and exhibited good internal reliability in the current study. Based on provided norms 43, *T*‐scores were calculated with higher scores reflecting higher endorsement of behavioural difficulties. Scores ≥ 64 were used as cut‐off to identify behavioural problems.

The Questionnaire to Assess Children's and Adolescents’ Emotion Regulation (FEEL‐KJ) 44 is a 90‐item measure to assess emotion regulation (ER) in children. Respondents indicate how frequently they endorse described strategies of ER on a 5‐point Likert scale ranging from 1 (‘almost never’) to 5 (‘almost always’). The FEEL‐KJ comprises 15 subscales and two second‐order scales. The second‐order scales adaptive ER (α = 0.93) with 42 items and maladaptive ER (α = 0.70) with 30 items were used and exhibited satisfactory to excellent levels of internal reliability in the current study. Based on provided norms 44, *T*‐scores were calculated with higher scores reflecting higher endorsement of ER strategies. Scores < 40 were used to identify deficient use of adaptive ER and scores > 60 to identify deficient use of maladaptive ER.

The Questionnaire of Resources in Children and Adolescents (FRKJ) 45 is a 60‐item measure to assess resources of children. Respondents indicate how strongly they agree with each item on a 4‐point Likert scale ranging from 1 (‘never true’) to 4 (‘always true’). In the current study, the subscale ‘self‐esteem’ with 6 items was used, exhibiting good internal reliability (α = 0.89). Based on provided norms 45, *T*‐scores were calculated with higher scores reflecting higher levels of self‐esteem.

Sociodemographic variables (age, gender, current school) were assessed with singular questions in self‐report form. Additionally, responsible caregivers in the foster care facility completed singular questions on the children's contact to parents, frequency of contact to parents, time since placement and household dysfunctions in the home of origin.

### Analysis

#### Confirmatory factor analysis

**Figure 1:**
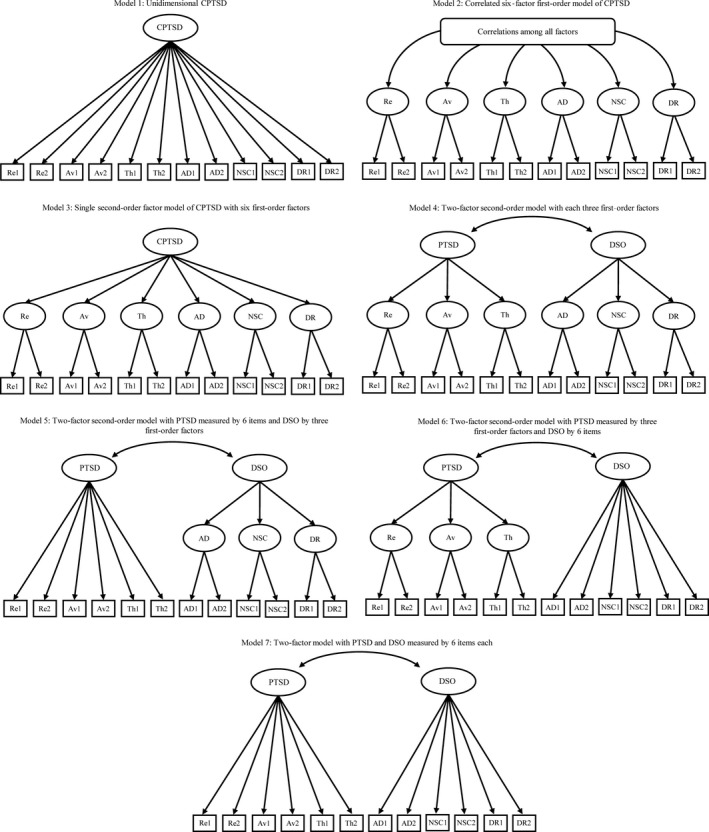
Seven alternative models of ICD‐11 CPTSD using the ITQ.

To test the symptom structure and factorial validity of ICD‐11 CPTSD in foster children, we conducted a series of confirmatory factor analyses (CFA). In accordance with past research on the factorial validity of ICD‐11 CPTSD in samples of adults 2, 3, 5, 10, seven alternative models were specified (Fig. 1). These models are hypothesized to resemble possible representations of PTSD and CPTSD according to ICD‐11, yet to be tested in children. Model 1 is a single‐factor model with all symptoms loading on a single latent variable (CPTSD). Model 2 is a six‐factor model with six correlated first‐order factors (Av, Re, Th, AD, NSC and DR). Model 3 comprises six first‐order factors and one single second‐order factor (CPTSD). Model 4 comprises six first‐order factors and two correlated second‐order factors (PTSD and DSO). Av, Re and Th load on the second‐order factor PTSD, and AD, NSC and DR load on the second‐order factor DSO. In Model 5, PTSD symptoms load directly on the PTSD factor, while DSO symptoms load on their respective first‐order factors (AD, NSC, DR), which load on the DSO factor. In Model 6, PTSD symptoms load on their respective first‐order factors (Av, Re, Th), which load on the PTSD factor, while DSO symptoms load directly on the DSO factor. In Model 7, all PTSD and DSO symptoms load directly on their respective factor (PTSD, DSO).

Each of these models was specified and tested in MPlus (version 7.3) 46 using the robust weighted least squares estimator (WLSMV). This estimator is based on the polychoric correlation matrix of latent continuous response variables and was identified as the most appropriate method of analysing ordinal indicators in a CFA context 47, producing correct parameter estimates, standard errors and test statistics 48. Missing data were managed using pairwise present analysis method, which is the default setting when using WLSMV estimator in Mplus 49. The amount of missing data on the ITQ was low with 0.0% to 2.2% missings on the item level.

Goodness of fit for each model was assessed using the Comparative Fit Index (CFI), the Tucker–Lewis Index (TLI) and the Root Mean Square Error of Approximation (RMSEA). Regarding CFI and TLI, values > 0.90 indicate adequate fit and values > 0.95 excellent fit 50, 51. Regarding RMSEA, values < 0.08 indicate adequate fit and values < 0.06 excellent fit 52. Since the WLSMV estimator does not produce information‐based indices, we also fitted the seven specified models using MLR 53 to generate Bayesian information criterion (BIC). Using BIC, nested and non‐nested models can be compared in regard to fit with lower values indicating better model fit 54. A 10‐point difference between two BIC values is strong evidence (odds ratio = 150:1) that the lower BIC model is statistically superior 55.

After the best‐fitting model was identified, factor scores and composite reliability (CR) were calculated. CR analysis calculates internal consistency of scales without the strict assumption of tau‐equivalence and is therefore recommended for measures with small numbers of items, like the ITQ. Values > 0.60 indicate acceptable internal consistency 56.

To investigate the discriminant validity of the ITQ scale scores based on the best‐fitting model of ICD‐11 CPTSD, summed PTSD and DSO scores were entered into a hierarchical regression model to predict 8 criterion variables comprising psychopathology (MDD, GAD, dissociation) and overall impairment (self‐esteem, behaviour problems, ER). In a first step, sociodemographic variables were entered into the regression model (gender, age, time in foster care, contact to parents, frequency of contact to parents), and in a second step, PTSD and DSO were added.

#### Latent class analysis

Latent class analysis (LCA) was conducted to identify homogenous classes of multivariate categorical data. First, binary variables were computed based on the cut‐offs of the ITQ to evaluate whether each of the 12 CPTSD symptoms was exhibited or not. Second, LCA was conducted to determine the number of classes based on the CPTSD symptoms. The general practice of LCA is to test the fit of a series of models, starting with a one‐class model, and to increase the number of classes until adding another class is no longer warranted. The fit of five models was assessed using MLR estimator. Avoiding solutions based on local maxima, 500 random sets of starting values and 100 final‐stage optimizations were used. Additionally, the maximum number of iterations allowed was set to 100. The relative fit of the calculated models can be compared using three information theory‐based fit indices: the Akaike information criterion (AIC), the BIC and the sample size adjusted BIC (aBIC). The model with the lowest values is deemed the best‐fitting model 54, 57, 58. In a simulation study, the BIC has been shown to be the best information criterion for identifying the correct number of classes 59. It is the most commonly used and trusted fit index for model comparison 60 and was thus chosen for the analysis. Additionally, the Lo–Mendell–Rubin adjusted likelihood ratio test (LMR‐A) 61 was used to compare models with increasing numbers of classes. A non‐significant *p*‐value (>0.05) suggests that the model with one less class should be accepted.

Chi‐squared tests and analyses of variance (ANOVAs) were performed to examine differences in sociodemographic characteristics, childhood trauma, psychopathology and overall impairment across the classes identified by the LCA.

## Results

### Descriptive statistics and diagnostic estimates

Overall, 48.5% of the sample experienced emotional abuse, 34.6% physical abuse, 28.7% sexual abuse, 50.7% emotional neglect, and 53.7% physical neglect. 58.1% of the sample reported multiple traumatization by having experienced more than one type of childhood trauma (cumulative childhood trauma). Regarding household dysfunctions, children experienced parents’ divorce (69.1%), substance abuse (32.4%), psychological disorders (30.1%), violence (29.4%) and criminal activities (13.2%) in their household of origin. 34.9% exhibited MDD, 26.4% GAD, and 28.1% dissociative symptoms. On a behavioural level, 44.9% showed internalizing and 22.8% externalizing behaviour problems. Regarding ER, 66.4% reported the use of adaptive strategies and 26.3% the use of maladaptive strategies. Functional impairment was reported in social interactions (36.0%), occupation (35.3%) and other important areas of life (48.5%).

### Confirmatory factor analysis

#### Factorial validity and psychometric properties

Model fit statistics for each model are presented in Table 1. Results showed that all models except for Model 1 showed excellent fit regarding CFI and TLI. Models 2, 4, 5 and 6 additionally showed acceptable fit regarding RMSEA. Of these four models, Model 4 and Model 5 yielded the lowest comparable BIC. Considering all indices together and the theoretical structure of ICD‐11 CPTSD, Model 4 was selected as the best‐fitting model as it combined high CFI (0.988) and TLI (0.984), low RMSEA (0.068; 95% CI = 0.039–0.095) and BIC (5165.742) and is in line with theoretical assumptions and previous findings. In order to check for stability of the results, model performance for all models was also examined using MLR estimator, whereas substantial results did not change (see supporting information).

**Table 1:** Model fit statistics for alternative models of ICD‐11 CPTSD (CFA) and latent class models (LCA)

Confirmatory factor analysis					
Model	χ^2^ (*df*)	RMSEA (90% *CI*)	CFI	TLI	BIC
1	166.137 (54) *	0.124 (0.102–0.145)	0.956	0.947	5254.526
2	59.221 (39) *	0.062 (0.025–0.092)	0.992	0.987	5185.724
3	106.429 (48) *	0.095 (0.070–0.119)	0.977	0.969	5186.498
**4**	**78.448 (48)** *	**0.068 (0.039**–**0.095)**	**0.988**	**0.984**	**5165.742**
5	88.430 (50) *	0.075 (0.049–0.100)	0.985	0.980	5164.648
6	92.976 (50) *	0.079 (0.054–0.104)	0.983	0.978	5178.185
7	102.518 (53) *	0.083 (0.058–0.107)	0.981	0.976	5177.813

Latent class analysis					
Model	Log likelihood (*df*)	BIC	Entropy	LMR‐A	Classes: *n*, %
1 class	−1032.173 (12)	2123.297	n.a.	n.a.	1: 136, 100%
2 classes	−904.325 (25)	1931.466	0.825	251.753 (*P *=* *0.026)	1: 52, 38.2%
					2: 84, 61.8%
**3 classes**	−**861.443 (38)**	**1909.568**	**0.873**	**84.441 (** *P** *** **=** *** *** **0.001)**	**1: 31, 22.8%**
					**2: 43, 31.6%**
					**3: 62, 45.6%**
4 classes	−842.359 (51)	1935.263	0.949	37.581 (*P *=* *0.040)	1: 16, 11.7%
					2: 30, 22.1%
					3: 30, 22.1%
					4: 60, 44.1%
5 classes	−828.325 (64)	1971.060	0.901	27.635 (*P *=* *0.133)	1: 27, 19.8%
					2: 30, 22.1%
					3: 30, 22.1%
					4: 32, 23.5%
					5: 17, 12.5%

*N *=* *136; estimator for CFA = WLSMV; estimator for LCA = MLR; CFA = confirmatory factor analysis; WLSMV = robust weighted least squares; LCA = latent class analysis; MLR = robust maximum likelihood; χ^2^ = chi‐squared goodness‐of‐fit statistic; *df* = degrees of freedom; RMSEA (90% CI)  = Root Mean Square Error of Approximation with 90% confidence interval; CFI = Comparative Fit Index; TLI = Tucker–Lewis Index; BIC = Bayesian information criterion; LMR‐A = Lo–Mendell–Rubin adjusted likelihood ratio test.

Best‐fitting model in bold.

*P <* *0.05.

Factor loadings for the selected model are reported in Table 2. The first‐ and second‐order factor loadings of PTSD and DSO were all positive and statistically significant (*P *<* *0.001). All PTSD first‐order factor loadings were high (>0.60) with the exception of one Th item ‘Being “super‐alert”, watchful or on guard’ (0.52). Similarly, all DSO first‐order factor loadings were high (>0.60), with the exception of one AD item ‘Taking a long time to calm down when upset’ (0.49). First‐order factors of Re, Av, Th, AD, NSC and DR loaded strongly onto their respective second‐order factor PTSD and DSO (>0.80 in all cases). PTSD and DSO were highly correlated (*r* = 0.75, *P *<* *0.001). The estimates of CR derived from the model estimates indicate excellent levels of internal reliability for the scale scores of PTSD (CR = 0.86) and DSO (CR = 0.91).

**Table 2:** Standardized factor loadings and standard errors for the two‐factor higher‐order model (Model 4)

Items	Re	Av	Th	AD	NSC	DR
Having upsetting dreams (Re 1)	0.75 (0.06)					
Having powerful images and memories (Re 2)	0.75 (0.06)					
Avoiding internal reminders (Av 1)		0.79 (0.06)				
Avoiding external reminders (Av 2)		0.70 (0.06)				
Being ‘super‐alert’, watchful or on guard (Th 1)			0.52 (0.08)			
Feeling jumpy or easily startled (Th 2)			0.76 (0.10)			
Long time to calm down when upset (AD 1)				0.49 (0.07)		
Feeling numb or emotionally shut down (AD 2)				0.68 (0.07)		
Feeling like a failure (NSC 1)					0.96 (0.02)	
Feeling worthless (NSC 2)					0.97 (0.02)	
Feeling distant or cut‐off from people (DR 1)						0.96 (0.06)
Finding it hard to stay emotionally close to people (DR 2)						0.63 (0.07)

First‐order factors	PTSD			DSO		
Re‐experiencing (Re)		0.88 (0.07)				
Avoidance (Av)		0.83 (0.08)				
Sense of current threat (Th)		0.80 (0.10)				
Affective dysregulation (AD)					0.99 (0.01)	
Negative self‐concept (NSC)					0.88 (0.04)	
Disturbances in relationships (DR)					0.92 (0.08)	

All factor loadings are statistically significant (*P *<* *0.001). *N* = 136.

PTSD, post‐traumatic stress disorder; DSO, disturbances in self‐organization.

#### Discriminant validity analysis

Results of hierarchical multiple regression analyses are reported in Table 3. Sociodemographic variables were entered in Step 1 and significantly contributed to explaining 6 of 8 criterion variables. In Step 2, PTSD and DSO were entered and significantly increased proportion of variance explained in all criterion variables (∆*R* ^*2*^ = 18–52%, *P *<* *0.001). The only model not significant at Step 2 included adaptive ER (*F*(7, 110)  = 2.080, *P* = 0.052) and was thus not considered in further analyses.

**Table 3:** Standardized coefficients for the regression model

	MDD	GAD	Dissociation	Self‐esteem	Internalizing behaviour problems	Externalizing behaviour problems	Adaptive ER	Maladaptive ER
Step 1 *R* ^*2*^	0.15***	0.12**	0.02	0.06*	0.09**	0.06*	0.00	0.27***
Gender	0.35***	0.28**	0.24*	−0.28**	0.32***	0.17	−0.14	0.50***
Age	0.11	0.12	−0.08	−0.11	0.08	0.04	0.00	0.08
Time in foster care	−0.06	−0.03	0.05	−0.04	−0.01	0.05	0.12	−0.03
Parents’ contact	0.11	0.18	0.02	0.01	0.03	0.24*	0.03	0.10
Frequency of parents’ contact	−0.03	−0.01	0.02	0.02	−0.08	−0.05	−0.05	0.00
Step 2 *R* ^*2*^ change	0.43***	0.41***	0.37***	0.18***	0.52***	0.19***	0.08**	0.19***
Gender	0.02	−0.04	−0.04	−0.09	−0.04	−0.03	0.01	0.29***
Age	0.07	0.08	−0.10	−0.03	0.02	0.03	0.05	0.05
Time in foster care	0.02	0.05	0.12	−0.11	0.02	0.06	0.07	0.06
Parents’ contact	0.13	0.20**	0.02	−0.04	0.05	0.23**	0.00	0.10
Frequency of parents’ contact	−0.06	−0.04	0.00	0.05	−0.10	−0.06	−0.03	−0.02
PTSD	0.21**	0.26**	0.35***	0.16	0.30***	0.27**	0.10	0.22*
DSO	0.60***	0.55***	0.42***	−0.55***	0.61***	0.27*	−0.38**	0.33**
Total variance explained	58.6%***	54.0%***	39.3%***	23.3%***	62.5%***	24.0%***	6.1%	45.5%***

MDD, major depressive disorder; GAD, generalized anxiety disorder; ER, emotion regulation; PTSD, post−traumatic stress disorder; DSO, disturbances in self‐organization.

*P <* *0.05. **P <* *0.01. ***P <* *0.001.

PTSD significantly predicted symptoms of dissociation (β = 0.35 (95% CI = 0.17, 0.53), *P *<* *0.001), internalizing behaviour problems (β = 0.30 (95% CI = 0.16, 0.43), *P *<* *0.001), externalizing behaviour problems (β = 0.27 (95% CI = 0.08, 0.47), *P *<* *0.01), GAD (β = 0.26 (95% CI = 0.11, 0.41), *P *<* *0.01), maladaptive ER (β = 0.22 (95% CI = 0.05, 0.40), *P *<* *0.05) and MDD (β = 0.21 (95% CI = 0.07, 0.36), *P *<* *0.01). DSO uniquely predicted self‐esteem (β = −0.55 (95% CI = −0.35, −0.80), *P *<* *0.001) and was a strong predictor for internalizing behaviour problems (β = 0.61 (95% CI = 0.46, 0.75), *P *<* *0.001), MDD (β = 0.60 (95% CI = 0.46, 0.78), *P *<* *0.001), GAD (β = 0.55 (95% CI = 0.40, 0.74), *P *<* *0.001) and dissociation (β = 0.42 (95% CI = 0.23, 0.63), *P *<* *0.001). DSO furthermore predicted maladaptive ER (β = 0.33 (95% CI = 0.14, 0.52), *P *<* *0.01) and externalizing behaviour problems (β = 0.27 (95% CI* *=* *0.06, 0.49), *P *<* *0.05). In order to control the stability of the results, criterion variables were also predicted using structural equation modelling, whereas substantial results did not change (see supporting information).

### Latent class analysis

Fit statistics of the LCA are reported in Table 1. The five‐class model did not yield a significant LMR‐A and was thus not considered for the final model. The two‐, three‐ and four‐class models all yielded significant LMR‐A (*P *<* *0.05). Of these three models, the three‐class model yielded the lowest BIC with a difference greater than 20 points, strongly supporting the statistical superiority of the three‐class model (BIC = 1909.568) over the four‐class model (BIC = 1935.263). Since the four‐class model also yielded significant LMR‐A (*P *=* *0.040), it was examined closely, but based on the fit indices, parsimony and the interpretability of symptom profiles, the three‐class model was selected.

**Figure 2:**
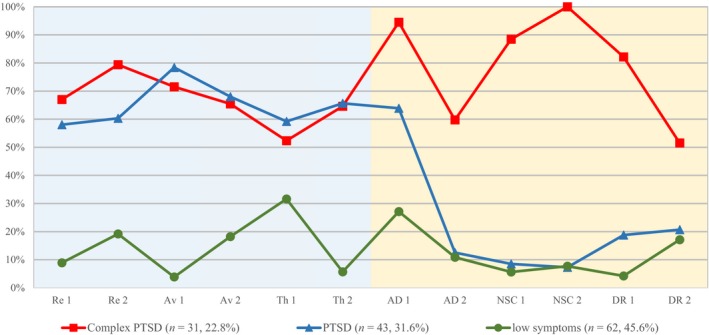
Endorsement of PTSD and DSO symptoms by class. [Colour figure can be viewed at http://wileyonlinelibrary.com]

The pattern of symptom endorsement of the three classes is presented in Fig. 2. To provide descriptive labels for each class, PTSD and DSO symptoms were compared among the three classes. Class 1 showed high levels of PTSD as well as DSO symptoms and was labelled ‘CPTSD’ (*n *=* *31, 22.8%). Class 2 showed high levels of PTSD but relatively low levels of DSO symptoms and was labelled ‘PTSD’ (*n *=* *43, 31.6%). Class 3 showed low levels of PTSD and DSO symptoms and was labelled ‘low symptoms’ (*n *=* *62, 45.6%). The mean probability of class membership for the CPTSD class was 0.972, for the PTSD class 0.958 and for the low symptoms class 0.920. An entropy value of 0.873 implies acceptable discrimination among the classes.

#### Sociodemographics, childhood trauma and symptom characteristics

The three classes did not differ regarding age (*F* (2, 132) = 1.549, *P *=* *0.216), time in foster care (*F* (2, 132) = 0.559, *P *=* *0.573), the foster care facility of residence (χ^2^(10) = 13.923, *P *=* *0.177), parents’ divorce (χ^2^ (2) = 0.026, *P *=* *0.987), substance abuse (χ^2^ (2) = 0.702, *P *=* *0.704) or delinquency of household members (χ^2^ (2) = 0.033, *P *=* *0.984). Differences were found regarding overall childhood trauma (*F* (2, 133) = 10.548, *P *<* *0.05) and domestic violence in the household of origin (χ^2^ (2) = 6.878, *P *<* *0.05) with significantly higher rates in the CPTSD class compared to the PTSD and low symptoms class. Further differences were found regarding psychiatric disorders of household members (χ^2^ (2)  = 11.165, *P *<* *0.05) with significantly higher rates in the CPTSD and PTSD class than the low symptoms class.

Detailed results of analyses comparing the three classes regarding further sociodemographics, forms of childhood trauma and symptom characteristics are provided in Table 4. No differences were found regarding nationality, contact to parents, physical abuse or physical neglect. However, the CPTSD class showed highest rates of emotional abuse, sexual abuse, emotional neglect, cumulative childhood trauma and proportion of females. The CPTSD and the PTSD class showed significantly higher rates of sexual abuse and cumulative childhood trauma than the low symptoms class.

**Table 4:** Sociodemographics, forms of childhood trauma and symptom characteristics across the three identified classes

Variables	Class 1 CPTSD (*n* = 31)	Class 2 PTSD (*n* = 43)	Class 3 low symptoms (*n* = 62)	Pairwise Post hoc comparison
Sociodemographics				
Females	74.2%	46.5%	24.2%	1 > 3
Austrian nationality	87.1%	90.7%	85.5%	n.a.
Contact to parents	96.8%	93.0%	93.5%	n.a.
Childhood trauma				
Emotional abuse	86.7%	50.0%	30.6%	1 > 2, 3
Physical abuse	38.7%	40.5%	30.5%	n.a.
Sexual abuse	48.4%	39.5%	11.3%	1, 2 > 3
Emotional neglect	74.2%	51.2%	40.0%	1 > 3
Physical neglect	61.3%	58.1%	47.5%	n.a.
Cumulative childhood trauma (> 1 type)	83.8%	67.4%	38.7%	1, 2 > 3
PTSD symptoms				
Upsetting dreams	67.7%	58.1%	11.5%	1, 2 > 3
Powerful images	80.6%	60.5%	21.0%	1, 2 > 3
Internal reminders	74.2%	83.7%	3.3%	1, 2 > 3
External reminders	64.5%	71.4%	19.4%	1, 2 > 3
Being super‐alert	51.6%	62.8%	31.1%	2 > 3
Feeling jumpy	64.5%	72.1%	4.8%	1, 2 > 3
DSO symptoms				
Long time to calm down	93.5%	65.1%	29.0%	1 > 2 > 3
Feeling numb	60.0%	11.9%	11.5%	1 > 2, 3
Feeling like a failure	90.0%	7.0%	6.5%	1 > 2, 3
Feeling worthless	100.0%	7.0%	8.2%	1 > 2, 3
Feeling distant	83.3%	18.6%	4.8%	1 > 2, 3
Hard to stay close	50.0%	20.9%	18.0%	1 > 2, 3
Comorbid disorders and symptoms				
MDD	78.6%	35.9%	14.5%	1 > 2 > 3
GAD	67.9%	30.8%	4.8%	1 > 2 > 3
Dissociation	62.1%	33.3%	8.3%	1, 2 > 3
Behaviour and ER				
Internalizing behaviour problems	87.1%	48.8%	21.0%	1 > 2 > 3
Externalizing behaviour problems	48.4%	20.9%	11.3%	1 > 2, 3
Adaptive ER	51.7%	69.4%	71.7%	n.a.
Maladaptive ER	72.4%	16.7%	10.0%	1 > 2, 3
Functional impairment				
Social	70.0%	37.2%	20.0%	1 > 2, 3
Occupational	61.3%	37.2%	21.0%	1 > 3
Other important areas	80.0%	65.1%	22.6%	1, 2 > 3

All tests were chi‐squared tests with 2 degrees of freedom; significance of all tests with reported post hoc comparisons was *P *<* *0.01; significant pairwise post hoc comparisons used adjusted *p*‐values using the Bonferroni correction.

PTSD, post‐traumatic stress disorder; DSO, disturbances in self‐organization; MDD, major depressive disorder; GAD, generalized anxiety disorder; ER, emotion regulation.

Consistent with the graphic depiction in Fig. 2, members of the CPTSD class showed significantly higher rates of PTSD symptoms than the low symptoms class and significantly higher rates of DSO symptoms than the PTSD and the low symptoms class. Members of the PTSD class showed similar rates of PTSD symptoms and lower rates of DSO symptoms than the CPTSD class. Members of the low symptoms class showed significantly lower rates of PTSD symptoms than the CPTSD and PTSD class and lower rates of DSO symptoms than the CPTSD class. Furthermore, members of the CPTSD class showed the highest rates of MDD, GAD, dissociation, internalizing and externalizing behaviour problems, maladaptive ER and functional impairment. Members of the PTSD class showed significantly lower rates of MDD, GAD, behaviour problems, ER and functional impairment. Members of the low symptoms class showed the lowest rates in all variables.

## Discussion

This study was conducted to examine the factorial and construct validity of ICD‐11 PTSD and CPTSD in children using the ITQ and consisted of three parts: (i) testing the factorial validity of ICD‐11 CPTSD; (ii) examining the psychometric properties and discriminant validity of ITQ scales; and (iii) testing the construct validity of ICD‐11 CPTSD.

Testing the factorial validity of ICD‐11 CPTSD, we identified the two‐factor higher‐order model (Model 4) as the best‐fitting model in children. PTSD and DSO were identified as correlated but distinct higher‐order factors, each comprising three first‐order factors, which resemble the symptom clusters of PTSD (Re, Av, Th) and DSO (NSC, AD, DR). These results are in line with the conceptualization of ICD‐11 CPTSD and findings in adults using the ITQ 2, 3, 8 as well as archival data 4, 5, 6. Also in line with previous studies 2, 3, 4, 5, 8, 10, the correlated six‐factor model (Model 2) yielded very good fit in our sample and identified as additional possible representation of CPTSD symptom structure in children. Nevertheless, because of its increased parsimony, better model fit and being in line with theoretical assumptions, Model 4 was chosen ultimately, supporting the factorial validity of ICD‐11 CPTSD and the distinction of PTSD and DSO in children.

Examining the psychometric properties of ITQ scales, our analyses revealed the ITQs’ applicability in children for the first time. All first‐ and second‐order factor loadings were statistically significant and high, and scores of the PTSD and DSO scales both showed excellent levels of internal reliability. Overall, only two items exhibited factor loadings < 0.60, one Th item ‘being “super‐alert”, watchful, or on guard’ and one AD item ‘taking a long time to calm down’. Since this is the first time the final version of the ITQ was applied in children and former studies in adult populations using previous ITQ versions did not report similar loadings 2, 3, we hypothesized that item formulations may be accountable for the lower loadings. As the two AD items comprise different facets of AD (hyperactivation: ‘taking a long time to calm down when upset’; deactivation: ‘feeling numb or emotionally shut down’) 8, lower loadings unto one factor may be explained by their content. Furthermore, the possibility of lower factor loadings because of understanding difficulties cannot be ruled out ultimately, despite the close instructions and guidance by the team of trained clinical psychologists during assessment.

The validity of PTSD and DSO as distinct ITQ scales was further evidenced by hierarchical regression analyses. PTSD significantly predicted dissociation, GAD and behaviour problems. DSO uniquely predicted self‐esteem and strongly predicted internalizing behaviour problems, GAD and dissociation. With the substantial variance explained in each criterion variable, first empirical support for the discrimination between PTSD and DSO in children was provided, in line with findings in adult populations 2, 8. Taken together, our results yield first empirical evidence that the ITQ is also applicable in children to assess ICD‐11 PTSD and CPTSD with accurately distinguishing between PTSD and DSO. Upcoming studies should further examine the ITQ scales properties in clinical as well as community samples of children. Structural equation modelling should be used to examine and replicate the current findings in larger samples.

The construct validity of ICD‐11 CPTSD was confirmed in children for the first time, capturing the symptom contents of ICD‐11 CPTSD precisely. Three groups of individuals with symptom profiles corresponding to CPTSD, PTSD and low symptoms emerged, in line with the ICD‐11 conceptualization and previous studies 16, 18, 19, 20. Further analyses revealed a coherent picture of sociodemographic characteristics, childhood trauma and comorbid disorders associated with PTSD and CPTSD. Children with CPTSD exhibited highest rates of childhood trauma, MDD, dissociation, GAD, behaviour problems, ER difficulties, functional impairment and the highest proportion of females. This is in line with previous findings in adult populations 18, 19, 20, 22, 23 and theoretical assumptions 17, 25. Children with CPTSD also exhibited highest rates of cumulative childhood trauma and significantly higher rates of cumulative childhood trauma than children with low symptoms. However, children with CPTSD did not show significantly higher rates of cumulative childhood trauma than children with PTSD, despite a non‐significant tendency in this direction (83.8% vs. 67.4%). This is in line with studies in adults, where CPTSD was repeatedly associated with highest rates of childhood trauma and other types of interpersonal traumatization, and partly with highest rates of cumulative childhood trauma and other types of multiple traumatization 18, 19, 62. In contrast to previous studies in children, where no differences regarding rates of depression, anxiety and number of comorbid disorders between PTSD and CPTSD were found 16, our results showed elevated rates of childhood trauma, comorbidity and impairment in children with CPTSD. Taken together, we found substantial evidence for the construct validity of ICD‐11 PTSD and CPTSD as empirically distinguishable disorders in children.

Regarding the under‐investigated population of children in foster care, our results show that foster children exhibit high rates of traumatization, psychopathology and complex trauma‐related disorders. With 22.8% of our sample falling in the CPTSD class, 31.6% in the PTSD class and 45.6% in the low symptoms class, our results confirm once more that foster children resemble more a clinical than a non‐clinical population 26. These findings evidence the need to include this vulnerable population of children stronger in trauma research and to screen for CPTSD routinely in children entering the welfare system.

The current study comprises several limitations. First, the size of the analysed sample of children in foster care is relatively small in comparison with studies conducted in adults, and even though our results confirm theoretical assumptions, current conclusions have to be considered with some caution. Replication using larger, clinical and community samples is needed. Second, a considerable portion of the original study sample (*n *=* *52, 36%) did not fill out the ITQ because of not having experienced any traumatic events according to self‐report. With subsequent analyses revealing excluded individuals being significantly younger and male with a higher tendency to minimize childhood trauma, current findings may deviate from true population effects. Since maltreated children may still feel a sense of loyalty to family members or may be unable to recall traumatic experiences accurately 63, 64, design inherent underreporting was expected to some extent, representing a limitation nonetheless. Third, no additional measure to assess PTSD was included, thus not allowing to examine the concurrent validity of ITQ scales. Lastly, our results substantiate the ITQs’ applicability in children, but the possibility of some understanding difficulties could not be ruled out ultimately, despite the close instructions and guidance by the team of clinical psychologists during assessment. Upcoming studies are needed to further test and optimize the ITQ in younger age groups.

In conclusion, the current study supports the factorial and construct validity of ICD‐11 CPTSD in children for the first time using precise descriptions of ICD‐11 symptom content. The symptom structure of ICD‐11 CPTSD was confirmed, supporting the distinction of PTSD and DSO as related but separate constructs. Thus, clinicians should not only screen for PTSD but also for DSO symptomatology in children, especially in children entering the welfare system or otherwise likely exposed to childhood trauma. Supporting the reliability and validity of ITQ scales, our findings provide first empirical evidence of the ITQs’ applicability as an easy‐to‐use screening instrument for ICD‐11 PTSD and CPTSD in children. Furthermore, our results substantiate that PTSD and CPTSD are two distinct disorders in children, associating CPTSD with highest rates of childhood trauma, comorbidity and functional impairment, further emphasizing the validity and clinical relevance of this distinction 14. Treatment intervention and duration of treatment may differ because of the nature, severity and comorbidity of PTSD and CPTSD symptoms in children, which should be subject of further research.

## Declaration of Interest

The authors declare that they have no competing interests.

## Supporting information


**Table** **S1**. Model fit statistics for alternative models of ICD‐11 CPTSD (CFA) using MLR estimator.
**Table** **S2**. Standardized coefficients for the structural equation model predicting criterion variables.

## Data Availability

Data subject to third party restriction: Data were assessed in the course of a research project commissioned and financed by the government of Lower Austria. Due to legal reasons, primary data cannot be shared.
