# Supplementary material for: Validation of ICD‐11 PTSD and complex PTSD in foster children using the International Trauma Questionnaire
Source: Acta Psychiatr Scand. 2019 Oct 16;141(1):60–73. doi: 10.1111/acps.13100 (PMC6973040; doi:10.1111/acps.13100)
Supplement: Supplementary file 1 — Table S1. Model fit statistics for alternative models of ICD‐11 CPTSD (CFA) using MLR estimator. Table S2. Standardized coefficients for the structural equation model predicting criterion variables. [file ACPS-141-60-s001.docx]

**Supporting Information**

| Table 1.  Model fit statistics for alternative models of ICD-11 CPTSD (CFA) using MLR estimator. | | | | | | |
| --- | --- | --- | --- | --- | --- | --- |
| **Confirmatory Factor Analysis** | | | | | | |
| **Model** | **χ^2^ (*df*)** | **RMSEA (90% CI)** | **CFI** | **TLI** | **SRMR** | **BIC** |
| 1 | 176.443 (54) * | .129 (.108 – .151) | .746 | .689 | 0.108 | 5254.526 |
| 2 | 55.948 (39) * | .057 (.014 – .088) | .965 | .940 | 0.050 | 5185.724 |
| 3 | 94.419 (48) * | .084 (.059 – .109) | .904 | .867 | 0.069 | 5186.498 |
| **4** | **70.183 (48) *** | **.060 (.027 – .088)** | **.952** | **.932** | **0.063** | **5165.742** |
| 5 | 81.296 (50) * | .068 (.039 – .094) | .935 | .914 | 0.065 | 5164.648 |
| 6 | 93.366 (50) * | .080 (.054 – .105) | .910 | .881 | 0.077 | 5178.185 |
| 7 | 105.222 (53) * | .085 (.061 – .109) | .892 | .865 | 0.078 | 5177.813 |
| *Note*. *N* = 136; Estimator for CFA = MLR; CFA = confirmatory factor analysis; MLR = robust maximum likelihood; χ^2^ = chi-square goodness of fit statistic; *df* = degrees of freedom; RMSEA (90 % CI) = Root-Mean-Square Error of Approximation with 90% confidence interval; CFI = Comparative Fit Index; TLI = Tucker Lewis Index; SRMR = Standardized Root Mean Square Residual; BIC = Bayesian Information Criterion.  Best fitting model in bold.  * *p* < .05. | | | | | | |

| Table 2.  Standardized coefficients for the structural equation model predicting criterion variables. | | | | | | | | |
| --- | --- | --- | --- | --- | --- | --- | --- | --- |
|  | **MDD** | **GAD** | **Dissociation** | **Self esteem** | **Internalizing behaviour**  **problems** | **Externalizing behaviour**  **problems** | **Adaptive**  **ER** | **Maladaptive ER** |
| **Step 1 *R^2^*** | .18** | .07 | .06 | .11* | .12* | .09 | .04 | .29*** |
| Gender | .35*** | .28** | .24** | -.28** | .32*** | .17 | -.14 | .50*** |
| Age | .19 | .12 | -.08 | -.11 | .08 | .05 | .01 | .09 |
| Time in foster care | -.06 | -.02 | .05 | -.05 | -.02 | .05 | .13 | -.01 |
| Parents contact | .10 | .17 | .02 | .01 | .03 | .24* | .03 | .09 |
| Frequency parents contact | -.03 | -.01 | .02 | .02 | -.08 | -.05 | -.05 | -.01 |
| **Step 2 *R^2^* change** | .49*** | .58*** | .51*** | .28*** | .64*** | .22*** | .11*** | .19*** |
| Gender | .02 | -.04 | -.04 | -.08 | -.05 | -.02 | -.01 | .30*** |
| Age | .07 | .09 | -.08 | -.01 | .03 | .05 | .05 | .07 |
| Time in foster care | .01 | .03 | .10 | -.12 | .01 | .05 | .08 | .05 |
| Parents contact | .12 | .18* | -.01 | -.06 | .03 | .21* | -.01 | .09 |
| Frequency parents contact | -.04 | -.02 | .01 | .03 | -.08 | -.05 | -.05 | -.02 |
| PTSD | .23 | .31* | .56*** | .33* | .39*** | .45** | .21 | .38* |
| DSO | .63*** | .53*** | .24 | -.77*** | .55*** | .09 | -.48** | .27 |
| Total variance explained | 67.6 % *** | 64.6 % *** | 57.2 % *** | 38.7 % *** | 76.2 % *** | 31.4 % *** | 15.0 % | 48.1 % *** |
| *Note.* MDD = major depressive disorder; GAD = generalized anxiety disorder; ER = emotion regulation; PTSD = posttraumatic stress disorder; DSO = disturbances in self-organization.  **p* < .05. ***p* < .01. ****p* < .001. | | | | | | | | |
